# The association between self-compassion and self-rated health in 26 samples

**DOI:** 10.1186/s12889-020-8183-1

**Published:** 2020-01-16

**Authors:** Fuschia M. Sirois

**Affiliations:** Department of Psychology, University of Sheffield, 1 Vicar Lane, Sheffield, S1 1HD UK

**Keywords:** Self-compassion, Self-rated health, Positive affect, Negative affect

## Abstract

**Background:**

Although there is growing evidence of the relevance of self-compassion for understanding health outcomes, few studies have examined self-compassion in relation to self-reported physical health status, also known as self-rated health (SRH). This study addressed this gap by examining the associations between self-compassion and SRH across multiple samples and after accounting for the contributions of positive and negative affect.

**Methods:**

Data from 26 samples (total *N* = 6127), comprised of 6 university student, 16 community adult, and 4 chronic illness samples, were included in the current analyses. Participants in each sample completed a survey including measures of self-compassion and SRH. Thirteen samples also completed a measure of positive and negative affect. The associations between self-compassion and SRH were statistically meta-analysed. Moderator analyses were conducted to test whether the associations varied as a function of sample type, age or participant sex. Semipartial correlations were calculated controlling for positive and negative affect in 13 samples and meta-analysed.

**Results:**

Findings indicated that self-compassion was significantly associated with higher SRH across the 26 samples (*r*_*avg*_ = .25; CI: .22, .28). The associations did not however vary significantly across sample types, or as a function of participant sex or age. The meta-analyses of the adjusted effects found that self-compassion remained significantly associated with higher SRH after accounting the contributions of positive (*sr*_*avg*_ = .11; CI: .07, .15) and negative (*sr*_*avg*_ = .25; CI: .06, .15) affect.

**Conclusions:**

The current study demonstrated that self-compassion is robustly associated with higher SRH across 26 samples and that this association remained significant after adjusting for the influence of positive and negative affect in 13 samples. Further longitudinal and experimental research is needed to verify the causal direction between self-compassion and SRH suggested by theory and the current findings.

## Background

A growing body of evidence has highlighted the significance of self-compassion for understanding health trajectories and outcomes. Defined as taking a kind, non-judgmental, connected, and mindful stance towards oneself during times of failure and difficulty [1], self-compassion has been linked to a number of consequential health-related outcomes that underscore its relevance for public health. Self-compassion is associated with lower levels of self-reported stress in medical and non-medical populations [2, 3], and attenuated unhealthy physiological responses to stress [4–6]. Evidence also supports the role of self-compassion for a variety of important health behaviours including exercise and healthy eating [7–9], sleep hygiene [10, 11], smoking cessation [12], self-care in medical populations [13], and medical adherence [14, 15]. Importantly lower stress and healthy emotion regulation have been identified as key explanatory pathways for the links to health behaviours [9, 14]. Given evidence supporting the protective role of self-compassion for reducing stress and for promoting health behaviours, and the known contributions of stress and health behaviours to physical health status [16], it is therefore reasonable to expect that self-compassion would be associated with better physical health. Yet to date, few studies have examined self-compassion in relation to physical health status.

Self-compassion has been conceptualized as including six key components organized along three bipolar dimensions [1], each of which can have benefits for health. Self-kindness versus self-judgement refers to responding to perceived inadequacy or difficulties with understanding, patience, and acceptance, rather than with harsh self-criticism. This response can defuse rather than perpetuate negative emotions and promote self-acceptance, which can down-regulate stress and thus be protective for health. Common humanity versus isolation refers to the recognition that all people are imperfect, make mistakes, and experience failure, rather than experiencing one’s shortcomings as unique or special, and thus feeling isolated by this egocentric perspective. By taking this broader and more connected perspective, self-compassionate people can more easily view their struggles in general, and with health issues and health behaviour changes in particular, as being part of the human condition. This can reduce the barriers to seeking help when in times of need [2, 3], and potentially improve health. Lastly, mindfulness refers to being aware of one’s current emotional states and suffering without becoming over-identified with the negative feelings that arise after failure or during struggles. This balanced mindset can minimize rumination over such failures and challenges including those that inevitably arise while trying to improve or manage one’s health. This in turn can free up self-regulation resources to support performance of behaviours to promote good health [17]. Together, these six components of self-compassion are proposed to operate in distinct and synergistic ways to promote a healthier way of responding to the inevitable failures and challenges of life [18], and thereby promote good health.

A burgeoning body of research supports the theoretical links between self-compassion and various factors that influence physical health. For example, self-compassion has been linked to lower levels of self-reported stress [3, 19], and to physiological markers indicating lower stress [4, 5]. With respect to the latter, research has found that self-compassionate individuals have lower sympathetic nervous system activation and reduced inflammatory response following exposure to a stressor [4–6], and higher heart rate variability, an index of parasympathetic influence on the heart that reflects greater ability to return to a resting state following acute stress [6, 20]. Evidence also indicates that self-compassion is associated with the practice of important health-promoting behaviours, such as exercise and diet, which are known to be modifiable risk factors for disease [21], and medical adherence, a key behaviour for health maintenance and disease prevention [22]. In a meta-analysis of 15 samples (*N* = 3252) self-compassion was positively associated with an index reflecting more frequent practice of a variety of health-promoting behaviours, including healthy eating and regular exercise [9]. Self-compassion has also found to be associated with better medical adherence across five medical samples including individuals with fibromyalgia, cancer, chronic fatigue syndrome [14], and better self-management behaviours in Type II diabetes [13]. Consistent with the theorized links between self-compassion and health, lower levels of negative affect and stress were found to explain in part why self-compassion people engaged in better health-promoting and health management behaviours, respectively [9, 14].

Despite the growing evidence base linking self-compassion to factors associated with better health, there is far less research on how self-compassion is linked to overall physical health. Self-rated health (SRH) is one reliable measure of overall physical health that is known to predict a number of objective measures of health status, including morbidity and mortality, health behaviours, serum high-sensitivity C-reactive protein, and cortisol responses to stress, even after accounting for other confounding factors [23–27]. SRH is captured via a single statement asking respondents to rate their health from poor to excellent, most commonly on a 5-point rating scale. A key distinction between SRH and other measures of physical health is that it is proposed to be a “summary statement about the way in which numerous aspects of health, both subjective and objective, are combined within the perceptual framework of the individual respondent” [28], p., 92. Importantly, numerous studies provide evidence that SRH is not only associated with current health status but also a predictor of future health (see Benyamin [29] for a review).

To date there have been few studies examining the link between self-compassion and SRH. Using composite measures of physical health that included the single item SRH, three studies have found that self-compassion was linked to better physical health among adult samples [7, 8, 30]. However, one study using an undergraduate sample found a small but significant negative association between self-compassion and a composite measure of physical health that included the global SRH item [31]. However, in each of these studies, factors known to attenuate or amplify perceptions of health were not accounted for. Given this, and the limited research to date, further research on how self-compassion relates to SRH with more diverse samples is warranted.

**Fig. 1:**
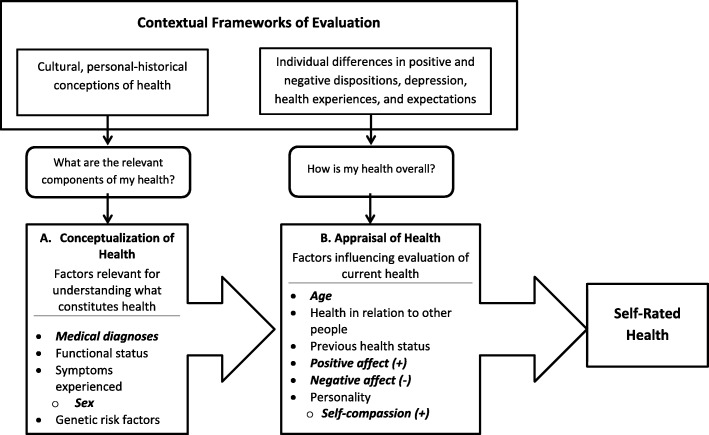
Operational model of the role of contextual factors in self-rated health as suggested by the Cognitive Process Model of Self-Rated Health [23], adapted from [14], and reproduced with permission. Boxed arrows represent the steps in the process of individual health evaluation rather than causal pathways. Bolded italic factors are those tested in relation to self-rated health in the current study

One potentially useful model for understanding why self-compassionate people may report better SRH is the Cognitive Process Model of SRH [23]. According to this model, answering the question of “How do you rate your current health?” involves an active cognitive process of reflection and self-assessment that necessarily takes places within a contextual framework that includes socio-cultural and individual differences. In particular, it highlights the role of personality as well as positive and negative affective states. Fig. 1 presents an operational model of the contextual factors that contribute to the process of evaluating one’s current physical health status as suggested by the Cognitive Process Model of SRH [23]. This multi-stage process begins with considering the relevant cultural and personal-historical information that can contribute to one’s health, including existing medical diagnoses and functional status, symptoms experienced, genetic risk factors, and biological sex. Of particular relevance for understanding how self-compassion relates to SRH, the next stage in the evaluation process involves appraising and summarizing this initial evaluation within the context of individual differences in positive and negative dispositions, age, previous health status, depression, health expectations and experiences. Together the evaluations from these processes inform the overall self-rating of health [23].

From the lens of the Cognitive Process model, there are several reasons to expect that self-compassion is associated with better SRH. Self-compassion reflects a way of relating to oneself in a positive manner when dealing with personal challenges and failures [1]. In this respect self-compassion is akin to other cognitive reappraisal processes that aim to reframe a situation to change the way it is emotionally responded to (Gross, 1998). Indeed, research has found that self-compassionate people tend to use cognitive reappraisal emotion regulation strategies to help reduce their negative mood [11, 32]. In the context of health challenges, self-compassionate people may therefore perceive their health status in a more positive light by being less critical of any health issues, viewing their health issues as part of the human condition, and reflecting on their health in a balanced rather than over-identified manner. Taken together the appraisals that self-compassionate people make towards their health suggest that they may evaluate their health status favorably. In addition, because SRH has strong associations with objective measures of health status [6, 23, 27], and self-compassion is associated with objective indicators of better health [4, 5], self-compassionate people may report better SRH in part because they experience fewer health symptoms, and thus have relatively better health compared to those with similar health profiles.

Although it is tempting to conclude that the link between self-compassion and SRH is due to the health protective nature of self-compassion as a positive quality, the high levels of positive affect and low levels of negative affect that characterize self-compassion [33] could also attenuate attention to physical states and symptoms and in this way result in higher ratings of SRH [34]. The Cognitive Process model posits that individual differences in positive and negative personality traits play a key role in shaping the evaluations that inform SRH, because levels of positive and negative affect, respectively, are known to attenuate or inflate attention to physical states and symptoms [35]. For example, personality traits linked to positive mood, such as conscientiousness and extraversion predict higher SRH, whereas traits linked to negative mood, such as neuroticism and self-critical perfectionism, are associated with lower SRH [35–39]. Following this line of reasoning, it is therefore important to control for positive and negative affect when understanding the extent to which self-compassion is associated with SRH.

Using the Cognitive Process Model of SRH [23] as a guiding framework, the aim of the current study was to address the limitations and inconclusive findings of previous research by providing a comprehensive analysis of whether self-compassion was associated with SRH across a large range of samples. In addition, the unique contribution of self-compassion to SRH was evaluated by controlling for positive and negative affect among a subset of samples that included a measure of positive and negative affect. Consistent with previous research indicating that self-compassion is associated with markers of good physical health [4, 6], it was hypothesised self-compassion would be positively associated with SRH. Self-compassion was further expected to be associated with lower levels of negative affect and higher levels of positive affect, as has been found in previous research [9, 33, 40]. Because the Cognitive Process model posits that SRH is determined by appraisals of current and past health that are based on objective health status, it was expected that self-compassion would remain significantly associated with SRH after accounting for the contributions of positive and negative affect to reflect the idea that self-compassion is linked to better overall health.

The first hypothesis was tested across a set of twenty-six samples including participants with a diverse range of health statuses, and the other hypotheses were tested among a subset of 13 samples from the 26 samples for which there were measures of positive and negative affect. For all sets of analyses, the associations were statistically meta-analyzed to estimate the magnitude of the effects (i.e., unadjusted and adjusted effects). This approach is recommended when findings are inconclusive and/or conflicting to help build a cumulative evidence base [41]. Summarising the associations this way also permitted a probing, through moderator analyses, of the contextual factors of health status (i.e., student, community adult, or medical sample), age and sex as suggested by the Cognitive Process Model [23], that might attenuate or amplify the magnitude of the proposed associations across different samples. Because research indicates that self-compassion may be particularly beneficial for health among individuals who have existing health problems [42], it was expected that the effects garnered from the chronic illness samples would be the largest relative to the community adult and student samples. The influence of sex and age on the associations of self-compassion with SRH were also examined in moderator analyses as both have been found to moderate the link between personality and health [23, 43].

## Methods

The present study included data from 26 independent samples (total *N* = 6127), comprised of 6 undergraduate and graduate student, 16 community adult, and 4 chronic illness (S8: arthritis, S9: inflammatory bowel disease, S10: chronic fatigue syndrome, S11: fibromyalgia) samples. Data were collected over a nine-year period from 2008 to 2017 as part of a larger research program focused on the dispositional correlates of health. Ethical clearance for the data collection was obtained through the respective Institutional Research Ethics Boards.

All samples except Sample 6 completed online surveys; accordingly, it was not possible to calculate response rates. The 16 community-dwelling adult samples were recruited from online and community sources, and the undergraduate and graduate student samples were recruited from two different post-secondary institutions. Samples were recruited using a variety of similar means including adverts posted on University volunteers lists, and notices posted on online psychology research websites, and on social media. Sample 6 was recruited with notices distributed via fitness instructors at their classes. Recruitment for the four chronic illness samples additionally utilised notices placed on relevant online support boards, and in the UK Fibromyalgia newsletter (S11). All participants gave consent prior to participating, and were given a chance to win gift cards of varying values as a participation incentive.

The demographic characteristics of each of the 26 samples are summarised in Table 1. Overall, the samples were predominantly white and female, except for Sample 26 which was all male.

**Table 1:** Demographic characteristics of the twenty-six samples

					Age (years)		Education level (%)		
Sample	*N*	Sample type	Percent female	Percent white	*M*	*SD*	High school	College/ university	Graduate school
1	162	Community	67.9	89.5	37.9	13.0	6.2	46.6	47.2
2	131	Community	75.9	75.2	30.4	11.8	8.9	68.9	22.2
3	117	Community	72.6	63.5	33.8	17.3	14.5	77.8	7.7
4	96	Community	84.8	83.8	33.9	11.4	6.7	61.9	31.4
5	637	Community	77.6	72.6	28.3	11.6	13.3	68.4	18.4
6	109	Community	73.9	88.7	31.07	15.7	7.2	79.3	13.5
7	104	Community	75.7	77.9	30.4	15.3	9.7	78.6	11.7
8	163	Chronic illness	91.4	92.7	46.9	11.5	13.3	66.1	20.6
9	155	Chronic illness	76.8	96.0	38.2	12.8	13.5	65.2	21.3
10	85	Chronic illness	84.7	93.7	35.3	14.7	11.8	61.2	27.1
11	165	Chronic illness	52.1	88.8	42.21	13.9	17.7	63.4	18.9
12	143	Community	77.0	87.6	29.5	13.0	19.6	27.3	53.1
13	236	Student	83.1	84.7	23.5	6.6	53.4	40.3	6.4
14	336	Student	82.0	73.8	21.7	4.9	44.7	47.9	6.3
15	189	Student	74.2	73.2	22.4	5.9	44.7	52.1	3.2
16	396	Student	75.1	85.8	21.20	4.1	–	100.0	–
17	289	Student	70.9	88.6	21.04	4.4	–	100.0	–
18	390	Student	85.8	75.1	21.20	4.1	–	100.0	–
19	102	Community	81.4	66.3	31.2	14.6	6.9	72.5	20.6
20	647	Community	30.3	85.6	30.6	12.3	8.4	51.5	40.1
21	341	Community	74.5	87.9	30.0	13.5	12.0	64.2	23.3
22	416	Community	78.4	84.5	29.0	10.8	7.7	55.3	37
23	159	Community	82.6	80.2	37.2	6.7	5.4	42.2	52.4
24	322	Community	100	85.0	24.3	5.4	7.5	58.1	34.5
25	90	Community	73.3	89.0	49.4	8.4	12.2	46.7	41.1
26	147	Community	0.00	74.1	24.4	5.6	6.1	59.2	34.7

### Measures

Participants completed standard demographic questions about age, gender, ethnicity, and education level. The means, standard deviations, and Cronbach alphas for all the scales are presented in Table 2.

**Table 2:** Summary of the characteristics of the study variables for the twenty-six independent samples

		Self-compassion			Negative Affect			Positive Affect			Self-Rated Health	
Sample	N	*M*	*(SD)*	α	*M*	*(SD)*	α	*M*	*(SD)*	α	*M*	*(SD)*
1	162	2.86	0.83	0.88	2.34	1.00	0.86	3.46	1.02	0.80	3.37	0.92
2	131	3.03	0.69	0.93	2.02	0.73	0.90	3.41	0.84	0.93	3.63	0.93
3	117	2.85	0.67	0.92	1.90	0.84	0.91	3.01	0.91	0.92	3.54	0.91
4	96	2.95	0.72	0.93	–	–	–	–	–	–	3.45	0.88
5	637	2.83	0.75	0.87	–	–	–	–	–	–	3.57	1.00
6	109	3.09	0.70	0.94	–	–	–	–	–	–	3.89	0.70
7	104	3.06	0.72	0.84	2.15	0.71	0.84	3.25	0.85	0.90	3.56	0.96
8	165	2.97	0.69	0.94	2.12	0.84	0.93	3.34	0.79	0.91	2.48	1.00
9	155	2.87	0.71	0.93	2.45	0.90	0.92	3.38	0.88	0.93	3.63	1.09
10	85	2.81	0.78	0.86	–	–	–	–	–	–	1.89	0.95
11	165	2.85	0.84	0.88	–	–	–	–	–	–	2.04	0.87
12	143	2.88	0.56	0.92	1.60	0.68	0.90	2.61	0.77	0.89	3.33	0.95
13	236	2.96	0.67	0.93	–	–	–	–	–	–	3.59	0.78
14	336	3.00	0.57	0.91	–	–	–	–	–	–	3.63	0.85
15	189	3.10	0.66	0.93	–	–	–	–	–	–	3.81	0.80
16	396	3.00	0.59	0.93	2.40	0.71	0.87	3.23	0.66	0.87	3.42	0.74
17	289	2.95	0.64	0.91	1.93	0.72	0.87	3.14	0.83	0.90	3.81	0.80
18	390	3.00	0.60	0.92	2.40	0.70	0.87	3.23	0.66	0.87	3.42	0.74
19	102	2.90	2.90	0.76	–	–	–	–	–	–	3.26	1.00
20	647	2.97	0.84	0.87	2.87	1.49	0.87	4.68	1.47	0.80	3.60	0.88
21	341	2.93	0.76	0.85	–	–	–	–	–	–	3.55	0.92
22	416	2.83	0.78	0.86	2.43	1.04	0.85	3.65	1.02	0.77	3.20	0.94
23	159	3.14	0.78	0.87	2.57	1.28	0.86	4.84	1.09	0.74	3.88	0.70
24	322	2.82	0.78	0.88	–	–	–	–	–	–	3.47	0.92
25	90	3.29	0.86	0.90	–	–	–	–	–	–	3.54	0.93
26	147	2.96	0.79	0.85	–	–	–	–	–	–	3.55	0.85

*Note: SD* Standard deviation

#### Self-compassion

Thirteen samples (Samples 2, 3, 4, 6, 8, 9, 12, 13, 14, 15, 16, 17, and 18) completed the 26-item Self-Compassion Scale [SCS; 44], and thirteen samples (1, 5, 7, 10, 11, 19, 20, 21, 22, 23, 24, 25, and 26) completed the short 12-item version of this scale [SCS-SF; 45]. The SCS assesses the three main components of self-compassion and their negative counterparts, Self-Kindness (Self-judgment), Common Humanity (Isolation), and Mindfulness (Over-identification). The SCS includes both positively (“I try to be loving towards myself when I’m feeling emotional pain”) and negatively (“I’m disapproving and judgmental about my own flaws and inadequacies“) worded items reflecting the six components of self-compassion. Research with diverse and international samples indicates that the subscales are best explained by a general overall factor of self-compassion [44]. Items are prefaced with the statement “How I typically act towards myself during difficult times” and respondents indicate how often they behave in the described way using response options ranging from 1 (Almost Never) to 5 (Almost Always). A total self-compassion score is calculated by averaging the mean subscale scores after reverse coding the negative items. Both the full SCS and the SCS-SF have been successfully used in both student and community samples, demonstrating good validity, both convergent and discriminate, and excellent test-retest reliability (α = .93) [45–47].

#### Self-rated health

Current SRH was assessed in all samples with the global health rating item from the Medical Outcomes Survey 36 item short form (SF-36) health questionnaire [48]. The SF-36 is a widely used, well-validated, and reliable measure of subjective health and overall physical well-being. The global health item asks participants “How do you rate your overall current health?” on a 5-point scale ranging from 1 (Excellent) to 5 (Poor). Responses are reverse scored so that higher values reflect better current SRH. The SF-36 global health item has demonstrated good criterion related validity, and is a predictor of several important health-related outcomes including, cortisol responses to stress, morbidity, and mortality [23, 24, 26].

#### Positive and negative affect

Thirteen of the 26 samples completed one of three versions of the Positive and Negative Affect Schedule PANAS [49]; to assess state negative and positive affect. Samples 7 and 12 completed the original 20 item PANAS which consists of 20 mood adjectives, 10 items of which assess state positive affect and 10 that assess state negative affect. Participants rate their current are rated on a 5-point Likert scale ranging from 1 for (*very slightly or not at all)* to 5 for (*extremely).* Samples 2, 3, 8, 9, 16, 17, and 18 completed the expanded 36-item PANAS X scale, which included the original PANAS items plus additional positive and negative affect adjectives. For consistency, only the items from the original 10 item negative affect and 10 item positive affect scales were used to calculate state negative and positive affect scores in these samples. Samples 1, 20, 22, and 23 completed a 10-item abbreviated version of the PANAS presented as a visual analogue scale, with 5 items for positive affect and 5 items for negative affect. Samples 1 and 22 rated items on 6-point scale with responses ranging from 1 (*very slightly or not at all)* to 6 for (*extremely).* Sample 1 rated items on an 8-point scale with options ranging from (*very slightly or not at all*) to 8 for (*extremely*), and Sample 23 rated items on a 7-point scale with options ranging from 1 (*very slightly or not at all)* to 8 for (*extremely).* The 20 item PANAS has demonstrated good discriminate and internal reliability (alpha = .88) [50].

## Analytic strategy

Data was analysed using SPSS version 23. Only cases that included a value for SRH were included in the analyses as this was a single item measure. The pattern of missing data was assessed using Little’s Missing Completely at Random (MCAR) test [51] in SPSS. If the test yielded a significant result indicating that the data were not missing completely at random, then missing data was imputed using multiple imputations [52]. This approach estimates the missing values by first imputing multiple (5) new sets of data with values for the missing cases. The values from the imputed data sets are then used to replace the missing values in the analyses that are conducted, and the results from the pooled values from the imputed data sets are used.

A multi-step approach was to examining the associations of self-compassion with SRH. The average unadjusted effect size of self-compassion with SRH across the 26 samples was estimated using a random effects model meta-analysis conducted with Comprehensive Meta-analysis (CMA), Version 2 software [53]. CMA transforms the individual correlation coefficients into Fisher’s *z* scores prior to meta-analyzing these effects. To understand the unique contribution of self-compassion to SRH beyond the contributions of positive and negative affect, the semi-partial correlations of self-compassion with SRH adjusted for positive affect and negative affect were calculated in the subset of 13 samples for which there positive and negative affect were measured. This yielded two sets of adjusted effects to meta-analyze. The magnitudes of the effect sizes were evaluated using Cohen’s standards [54], whereby *r* = .10 is considered a small sized effect, *r* = .30 is considered a medium sized effect, and *r* = .50 is considered a large sized effect.

To understand the associations of self-compassion to positive and negative affect, and of positive and negative affect to SRH, the corresponding correlations were calculated and then statistically meta-analyzed for the 13 samples that included measures of positive and negative affect. To assess any potential biases in the results for the adjusted effects obtained from analyzing half of the samples, a moderator analysis was also planned to compare the unadjusted effects among the samples that did and did not include a measure of positive and negative affect.

The variability in effect sizes between samples was evaluated with two approaches to determine whether the planned subgroup moderator analyses were warranted. First, the heterogeneity statistic, *Q*, assessed the degree of variability among the pool of effects sizes [55]. Moderator analysis is warranted if this statistic is associated with a large confidence interval. Second, the *I*^*2*^ statistic was used to estimate the proportion of variability present that is not due to sampling error within studies [56]. As a general rule, *I*^*2*^ values of 25% reflect low heterogeneity, 50% reflect moderate heterogeneity, and 75% or more reflect high heterogeneity [55].

Moderator analyses were planned to test the role of sample type (community vs. chronic illness vs. student), age, and sex, on the unadjusted and fully adjusted effects for both positive and negative affect. These analyses were only conducted if subgroups included three or more studies in line with Card’s (2102) caution regarding the reduction of statistical power and difficulties in detecting meaningful group differences when there are too few studies in a subgroup. Moderator analyses were conducted with a mixed effects approach where the combined subgroups were first analyzed with a random effects model to further assess heterogeneity within each subgroup, and then combined using a fixed effects model to assess the heterogeneity between subgroups. A mixed effects meta-regression (method of moments) analysis was used to assess the potential moderating effects of age and gender, as age was recorded as a continuous variable, and sex recorded as the percentage of the sample that was female.

To estimate of the number of studies with null results that would have to be included in the meta-analysis to render the current findings non-significant, a Failsafe *N* was calculated [57]. Accordingly, the fail-safe *N* was only calculated for those effects that reached statistical significance (*p* < .05). Rosenthal’s (1979) guidelines were followed for determining an adequately high fail-safe *N.* Accordingly*,* the Fail-safe N should be greater than 5 *k* + 10, where *k* = the number of studies included. Although all of the data sets meta-analyzed in the current study were unpublished, it was still important to calculate the Failsafe *N* because there were a relatively small number of samples included in the analysis, and because other researchers were not contacted to obtain other unpublished studies.

## Results

### Preliminary analyses

As the MCAR test was significant for at least one variable in the majority of the data sets a multiple imputation approach was taken to replace missing data for all data sets. Overall, the percentage of missing data across all 26 data sets was relatively low ranging from 0.5% to a high of 10.8% for self-compassion scale.

The results for the meta-analysed unadjusted and adjusted effects for self-compassion in relation to SRH, and the meta-analysed associations of positive and negative affect to self-compassion and SRH, are presented in Table 3.

**Table 3:** Meta-Analyzed Unadjusted (Across 26 Samples, Total *N* = 6127), and Adjusted Effects (Across 13 Samples, Total *N* = 3272), Among Self-Compassion (SC), and Self-Rated Health (SRH), Adjusting for Positive and Negative Affect

Sample	*N*	SC-SRH *r*	SC-PA *r*	SC-NA *r*	PA-SRH *r*	NA-SRH *r*	SC-SRH *sr*_*PA*_	SC-SRH *sr*_*NA*_
1	162	.333	.434	−.464	.391	−.345	.149	.162
2	131	.357	.613	−.522	.595	−.351	−.097	.161
3	117	.217	.129	−.223	.353	−.278	.168	.152
4	96	.203	–	–	–	–	–	–
5	637	.261	–	–	–	–	–	–
6	109	.526	–	–	–	–	–	–
7	104	.166	.498	−.620	.197	−.209	.066	.033
8	163	.050	.322	−.465	.260	−.369	−.037	−.135
9	155	.331	.543	−.643	.436	−.379	.068	.068
10	85	.200	–	–	–	–	–	–
11	165	.304	–	–	–	–	–	–
12	143	.248	.286	−.320	.188	−.237	.194	.170
13	236	.247	–	–	–	–	–	–
14	336	.278	–	–	–	–	–	–
15	189	.270	–	–	–	–	–	–
16	396	.258	.427	−.487	.281	−.182	.133	.168
17	289	.284	.319	−.396	.229	−.266	.209	.175
18	390	.260	.429	−.489	.281	−.181	.134	.170
19	102	.181	–	–	–	–	–	–
20	647	.254	.480	−.520	.304	−.340	.101	.066
21	341	.097	–	–	–	–	–	–
22	416	.235	.350	−.454	.300	−.353	.125	.064
23	159	.225	.399	−.516	.253	−.307	.121	.059
24	322	.137	–	–	–	–	–	–
25	90	.224	–	–	–	–	–	–
26	147	.319	–	–	–	–	–	–
Meta-analysis results	Effects 95% CI	.247 [.22, .28]	.409 [.35, .46]	−.478 [−.52, −.43]	.312 [.26, .36]	−.291 [−.33,-.25]	.113 [.07, .15]	.105 [.06, .15]

*Note*: *CI* Confidence interval

### Self-compassion and self-rated health

The meta-analysis of the effects for the 26 samples revealed a significant and positive small to moderate sized average association between self-compassion SRH when not accounting for the contributions of positive or negative affect (see Table 3). The test of heterogeneity revealed there was a significant amount of unexplained variability among the unadjusted effect sizes, *Q* (25) = 39.7, *p* < .05; *I*^*2*^ = 37.01%, indicating that the planned moderator analyses were warranted.

### Self-compassion, positive affect, and self-rated health

Across the 13 samples (total *n* = 3272) that included a measure of state positive affect, the meta-analysis revealed the expected positive associations between self-compassion and positive affect, and between positive affect and SRH. The meta-analysis of the effects of self-compassion and SRH, accounting for the contributions of positive affect found that self-compassion remained on average, positively and significantly associated with SRH, although the magnitude of the average adjusted effect was reduced to a small effect compared to the small to moderate sized effect for the unadjusted average effect. The tests of heterogeneity revealed a non-significant and low degree of variability among the effects *Q* (12) = 14.7, *p* = .26; *I*^*2*^ = 18.20%, indicating that moderation tests were not necessary.

### Self-compassion, negative affect, and self-rated health

Across the 13 samples, the analysis found the expected significant and negative associations between self-compassion and negative affect, and between negative affect and SRH. The analysis of the effects of self-compassion and SRH, adjusted for negative affect, revealed that self-compassion remained on average, negatively and significantly associated with SRH with a small sized effect. Similar to the adjusted effects for positive affect, the magnitude of the average adjusted effect was roughly half that of the unadjusted average effect. As well, the tests of heterogeneity were non-significant, indicating a low degree of variability among the effects, *Q* (12) = 18.7, *p* = .10; *I*^*2*^ = 35.80%. Accordingly, moderator tests were not conducted.

### Moderator analyses

The first moderator analysis assessed whether the magnitude of the unadjusted effects among the 13 samples that tested the effects adjusted for positive and negative affect varied significantly from the 13 samples that were not included in the analyses of the adjusted effects. The tests of heterogeneity were non-significant, *Q* (1) = 0.03, *p* = .87; *I*^*2*^ = 37.10%, indicating that the effects of self-compassion and SRH garnered from the 13 studies that tested the adjusted effects (*r*_*avg*_ = .252, 95% CI [.22, .29]) were not significantly different from those that were not included in these analyses (*r*_*avg*_ = .247, 95% CI [.19, .30]).

The next moderator analysis focused on whether the type of sample explained the heterogeneity in the unadjusted effects. The sub-group analysis of the unadjusted effects of self-compassion with SRH as a function of sample type was non-significant, *Q* (2) = 0.65, *p* = .72. The effects garnered from community samples (*r*_*avg*_ = .244; *k* = 16; 95% CI [.20, .29]) were not significantly different than those garnered from the chronic illness (*r*_*avg*_ = .225; *k* = 4; 95% CI [.09, .35]) and student samples (*r*_*avg*_ = .266; *k* = 6; 95% CI [.22, .31]).

The next moderator analyses focused on the role of age. The meta-regression testing the potential influence of participant age on the unadjusted effects for self-compassion and SRH was non-significant for the unadjusted correlations, *b* = − 0.00 [−.01, .00], *Q*_*model*_ (1) = 0.54, *p* = .46, *Q*_*residual*_ (24) = 26.05, *p* = .35.

The final moderator analysis examined the influence of participant sex on the associations of PC with SRH. The results of the meta-regression were not significant, *b* = − 0.14 [−.31, .03], *Q*_*model*_ (1) = 2.68, *p* = .10, *Q*_*residual*_ (24) = 25.15, *p* = .40. This indicated that the percentage of females in the samples did not have a significant influence on the magnitude of the associations between self-compassion and SRH.

### Failsafe *N* test

The Failsafe *N* analysis of the unadjusted effects of self-compassion and SRH indicated that an additional 2363 studies with non-significant results would need to be included in the set of studies that were statistically meta-analysed to reduce the *p* value below .05. This Failsafe *N* was well above the 5 *k* + 10 studies cutoff (140) recommended by Rosenthal (1979).

## Discussion

The current study provides the most comprehensive analysis of the relationship of self-compassion and SRH to date. Across 26 samples comprised of community adults, university students, and individuals with chronic illness, analysis revealed a small to moderate sized association of self-compassion with higher SRH. Consistent with the hypotheses, self-compassion remained, on average, significantly associated with better SRH after adjusting for the contributions of positive and negative affect in the 13 samples analysed. However, the magnitudes of the adjusted effects were reduced in size from the unadjusted effects. The subgroup analysis of the unadjusted effects revealed that the associations of self-compassion with SRH did not vary significantly as a function of sex, age or the sample type supporting the robustness of self-compassion for SRH across diverse samples.

A growing evidence base supports self-compassion as an important epidemiological factor for understanding health trajectories and outcomes. The current findings add to this research in several important ways. A significant limitation within previous research on self-compassion and health is that there have been few studies examining self-compassion in relation to self-rated health, a reliable measure of health status. Among those studies that have, results were inconsistent perhaps owing to the different samples examined (e.g., adult versus student samples). In addition, the potential contributions of positive and negative affect were not controlled for in previous studies examining self-compassion and SRH. The current research addressed these important gaps by finding that in 26 diverse samples, self-compassion was associated with SRH, a robust and reliable measure of physical health status that is linked to a variety of objective measures of physical health [23]. In this respect, the current findings extend previous evidence that self-compassion is linked to subjective and objective markers of stress [6, 19, 58], and health-promoting and health maintenance behaviours [9, 13, 14], by finding that self-compassion is also associated with SRH, a global measure of physical health.

By statistically meta-analysing the associations across the samples in the current study, it was possible to test the extent to which the associations between self-compassion and SRH varied as a function of contextual variables posited to influence SRH by the Cognitive Process model [23]. The meta-analysis of the unadjusted effects suggested that there was moderate and significant degree of variability. However, the subgroup analyses revealed that the type of sample, which was used as a proxy for health status, did not significantly affect the size of the associations garnered across samples. Previous research indicates that self-compassion may be particularly beneficial for health among individuals who have chronic health problems [42]. However, as there were only 4 chronic illness samples included in the analyses, the current findings need to be interpreted with caution as subgroup analyses tend to become less reliable as the subgroup size becomes smaller [55]. The moderator analyses of participant sex and age were also non-significant in the current study, despite previous theory and research suggesting that these contextual factors may moderate link between personality and health [23, 43].

Previous research on self-compassion and physical health has not accounted for the role of affective states, which are known to confound self-reports of health [34]. Using a theory driven approach, the current research examined the associations of self-compassion in relation to SRH after adjusting for the contributions of positive and negative affect. The results were consistent with the Cognitive Process Model [23] in that the adjusted effects were smaller in magnitude than those that did not account for positive and negative affective states. These findings suggest that the association between self-compassion and SRH can be explained in part by the high levels of positive affect and the low levels of negative affect that characterise self-compassion.

Although self-compassion can arguably be viewed as an affective state, the current findings support the notion that it is not simply a form of high positive affect and low negative affect. Rather, self-compassionate responding reflects a process of transforming and harnessing the negative affect experienced during suffering into a positive affective state, and specifically compassion, and as a means to motivate self-improvement [1]. Consistent with this view, self-compassion remained on average significantly, although modestly, associated with SRH after accounting for positive and negative affect, despite the medium-sized average association between self-compassion and higher positive affect, and lower negative affect. These findings make an important theoretical contribution to understanding how self-compassion is related to SRH by demonstrating that the links between self-compassion and SRH are not simply an artefact of affect-related reporting biases, and that self-compassion is more than simply an affective state.

In addition to the theoretical contributions for understanding how self-compassion relates to physical health, the current findings also have important practical implications. Self-compassion is quality that can be cultivated through training programs such as the Mindful Self-Compassion program [59], Compassionate Mind Training [60], and Compassion-Focused Therapy [61]. In addition to these group-based interventions, there are also kindness-based meditations and formal daily practices for individuals that can be effective for increasing self-compassion [62]. The robust link between self-compassion and SRH found in the current study provides promising initial evidence to support the proposition that changes in self-compassion might lead to changes in SRH. Given this, such training programs might be especially important for individuals living with chronic health conditions to improve their subjective evaluations of their health. This in turn could foster greater engagement with health maintaining behaviours such as medical adherence, as well as self-care behaviours including managing stress, following appropriate dietary and exercise recommendations [42], all of which can contribute to improvements in objective health status and overall quality of life [63]. Although the unadjusted effects were small to moderate, and the adjusted effects were small in magnitude, even small increases in SRH as a result of self-compassion training can still be considered important when viewed from a public health perspective that highlights the benefits of small changes over large numbers of individuals [64].

The current findings, though novel, should be considered in light of several limitations. The samples used in the analyses, although reflecting diverse populations, were convenience samples and therefore may not be representative of the wider populations from which they were drawn. It is therefore unclear as to whether similar findings would be obtained with more representative samples. Nonetheless, including 26 samples with over 6000 participants provides some initial evidence for a robust relationship between self-compassion and SRH. The current study used an aggregated data (AD) meta-analysis rather than an individual participant data (IPD) approach. It is therefore possible that the findings from a IPD analyses may differ from those obtained in the current study. However, the choice was made based on limited available resources to conduct IPD analyses, and previous work that found that the same data subjected to AD and IPD yielded estimates that were roughly the same for main effects [65].

The cross-sectional designs of the studies analysed preclude making any firm conclusions about the direction of association between self-compassion and SRH. For example, it is possible that it is easier to be self-compassionate when one has better SRH. However, according to theory, self-compassion is activated and therefore most beneficial when people experience difficulties and suffering, helping them to manage their suffering [1]. Research demonstrating the benefits of self-compassion for coping with chronic and painful health conditions such as arthritis, chronic pain, and inflammatory bowel disease [2, 40], supports the idea that self-compassion leads to better SRH. In addition, the assumed direction of the relationship in the current study is consistent with the Cognitive Process Model of SRH [23], which implicates individual differences such as self-compassion, in evaluations of overall health status. The proposed directionality from self-compassion to SRH is also in agreement with experimental and longitudinal research demonstrating the effects of self-compassion on other health-related outcomes including physiological responses to stress, and medical adherence [4, 5, 15, 66]. From these theoretical perspectives, the assumed temporal precedence from self-compassion to SRH makes more sense than the reverse. Nonetheless, longitudinal and experimental research is needed to confirm the directionality of the links between self-compassion and SRH assumed in the current research. Ostensibly, the use of a single item to assess physical health status could be seen as a limitation of the current research, as multi-item measures are often viewed as having better psychometric properties. However, research testing multi-item versus single item measures has indicated that this is not always the case, and that in some instances a single item measure can be as good or even more appropriate than multi-item measures of a construct [67]. Moreover, a large body of research demonstrating that SRH is a robust predictor of current and future health supports its validity as a single item measure of physical health [23, 29].

A notable strength of the current research is the demonstration of consistent associations between self-compassion and higher SRH across 26 diverse samples that overall show a small to moderate effect size. This increases confidence that the results will replicate. This comprehensive analysis also provides more conclusive evidence regarding how self-compassion is linked to SRH given the inconsistent findings in the small number of studies that previously examined the association of self-compassion to SRH. In addition, by situating this association within the conceptual framework of the Cognitive Process model of SRH [23], the current research highlights the potential cognitive and physical benefits of self-compassion for evaluations of health that go beyond the known affective benefits of taking a compassionate stance towards one’s short-comings and suffering.

## Conclusions

The current research makes an important contribution to our understanding of the potential benefits of self-compassion for physical health by finding that self-compassion was associated with higher SRH across 26 diverse samples, and that this average association remained significant when adjusted for the contributions of positive and negative affect in separate analyses with 13 samples. Importantly, the associations between self-compassion and SRH did not vary as a function of sample type, participant sex or age. Longitudinal and experimental research is needed to verify the causal direction between self-compassion and SRH suggested by theory and the current findings to gain further insights into the role of self-compassion for physical health. Such research, if found to support a temporal link from self-compassion to SRH, would be particularly valuable for informing interventions targeted at improving physical health in both general and clinical populations.

## Data Availability

The datasets used and/or analysed during the current study are available from the corresponding author on reasonable request.

## Abbreviations

CI: Confidence interval
SRH: Self-rated health
